# New archidermapteran earwigs (Dermaptera) from the Middle Jurassic of Inner Mongolia, China

**DOI:** 10.3897/zookeys.1065.72720

**Published:** 2021-10-26

**Authors:** Shurong Xiong, Michael S. Engel, Lifang Xiao, Dong Ren

**Affiliations:** 1 College of Life Sciences and Academy for Multidisciplinary Studies, Capital Normal University, 105 Xisanhuanbeilu, Haidian District, Beijing 100048, China Capital Normal University Beijing China; 2 Division of Entomology, Natural History Museum, and Department of Ecology & Evolutionary Biology, University of Kansas, 1501 Crestline Drive – Suite 140, Lawrence, Kansas 66045-4415, USA University of Kansas Lawrence United States of America; 3 Division of Invertebrate Zoology, American Museum of Natural History, Central Park West at 79th Street, New York, New York 10024-5192, USA American Museum of Natural History New York United States of America

**Keywords:** Dermapteridae, earwigs, new genus, new species, Protodiplatyidae, systematic palaeontology

## Abstract

Two new species of Archidermaptera are described and figured from the Middle Jurassic Jiulonghsan Formation of Daohugou, Inner Mongolia, China. *Aneurodermaoiodes***gen. & sp. nov.** is described in the family Protodiplatyidae and *Sinopalaeodermataconcavum***sp. nov.** is established in the family Dermapteridae. Both new species share the typical characters of the extinct suborder Archidermaptera (e.g., pentamerous metatarsi, filiform and multimerous cerci, externalized ovipositor). *Aneuroderma***gen. nov.** is compared with other genera of the Protodiplatyidae, while *S.concavum***sp. nov.** allows us to emend the diagnosis of the genus *Sinopalaeodermata*. We briefly discuss the diversity of Archidermaptera and challenges to understanding relationships among this mid-Mesozoic diversity.

## Introduction

The Dermaptera (earwigs) are, like all organisms, an interesting mosaic of primitive and derived traits – on the one hand they have a typical ‘orthopteroid’ habitus with chewing mouthparts, while on the other extant species have specialized cerci modified as forceps; a vestigial ovipositor; reduced tarsal count; a shortened and tegminized forewing; a unique hind wing composed of a greatly enlarged anal fan, reduced remigium, and distinctive folding pattern; and have lost ocelli (Haas 1995; Grimaldi and Engel 2005). Dermaptera also have specialized maternal care widespread through the order, and date to at least the Early Cretaceous, and perhaps the latest Jurassic (Engel 2009). While the order is of unexceptional diversity in terms of species numbers, with only about 2000 extant species, there is considerable morphological variety, particularly in the form of the thoraces, tegmina, abdomens, and cerci (Haas 1995; Sakai 1996; Grimaldi and Engel 2005). The order is of modest age, with crown-group representatives extending to the Early Cretaceous, and the clade as a whole extending to the Triassic when considering stem groups (Bey-Bienko 1936; Vishniakova 1980; Carpenter 1992). It is possible that the extinct order Protelytroptera are further stem-Dermaptera, extending the combined lineage into the Early Permian (Haas and Kukalová-Peck 2001; Grimaldi and Engel 2005).

Excluding the Protelytroptera, the earliest definitive earwigs are classified in the suborder Archidermaptera, distinguished from other suborders by the pentamerous metatarsi, frequent presence of venation in the forewing tegmina, which are often longer, and a prominent externalized ovipositor (Engel 2003). In addition, species plesiomorphically retain ocelli and usually long, multiarticulated cerci that are, of course, not forcipate. In addition, the pro- and mesotarsi are more developed than their counterparts of the Eodermaptera and Neodermaptera, with 4 or 5 tarsomeres rather than the trimerous condition of more derived clades. Presently, there are three families recognized within the family: Protodiplatyidae, Turanoviidae, and Dermapteridae (Engel 2003; Engel and Haas 2007). Hitherto, there have been 31 species classified in 19 genera, with specimens being comparatively rare (Xing et al. 2016a; Xing et al. 2016b; Tihelka 2019). Current records are from the Late Triassic of England, Australia, and Kyrgyzstan; the Middle Jurassic of China; the Late Jurassic of Kazakhstan; and the Early Cretaceous of China (Martynov 1925; Bey-Bienko 1936; Vishniakova 1980; Zhang 1994, 2002; Jarzembowski 1999; Wappler et al. 2005; Shcherbakov 2008; Zhao et al. 2011) (Table 1).

**Table 1.** Hierarchical classification of Archidermaptera and summary of tarsal formulae, where known. Interrogative marks (?) indicate missing data.

			Genus	Species	Data
Suborder Archidermaptera Bey-Bienko, 1936	Superfamily Protodiplatyoidea Martynov, 1925	Family Dermapteridae Vishniakova, 1980	Genus *Brevicula* Whalley, 1985	*B.gradus* Whalley, 1985	4/5-4/5-5*
				*B.maculata* Kelly et al., 2018	?-?-?
			Genus *Dacryoderma* Engel, 2021	*D.teres* (Tihelka, 2019)	?-?-?
			Genus *Dermapteron* Martynov, 1925	*D.incerta* Martynov, 1925	?-?-?
			Genus *Dimapteron*Kelly et al., 2018		
				*D.corami* Kelly et al., 2018	?-?-?
			Genus *Jurassimedeola* Zhang, 2002	*J.orientalis* Zhang, 2002	?-?-?
			Genus *Palaeodermapteron*Zhao et al., 2011	*P.dicranum* Zhao et al., 2011	5-5-5
			Genus *Phanerogramma* Cockerell, 1915	*P.australis* Kelly et al., 2018	?-?-?
				*P.dunstani* Kelly et al., 2018	?-?-?
				*P.gouldsbroughi* Kelly et al., 2018	?-?-?
				*P.heeri* (Giebel, 1856)	?-?-?
				*P.kellyi* Tihelka, 2019	?-?-?
			Genus *Sinopalaeodermata* Zhang, 2002	*S.concavum* sp. nov.	5-5-5
				*S.neimonggolense* Zhang, 2002, nom. emend.	5-5-5
			Genus *Trivenapteron*Kelly et al., 2018	*T.moorei* Kelly et al., 2018	?-?-?
			Genus *Valdopteron*Kelly et al., 2018	*V.woodi* Kelly et al., 2018	?-?-?
		Family Protodiplatyidae Martynov, 1925	Genus *Abrderma*Xing et al., 2016b	*A.gracilentum* Xing et al., 2016b	?-?-5
			Genus *Aneuroderma* gen. nov.	*A.oiodes* sp. nov.	5-5-5
			Genus *Archidermapteron* Vishniakova, 1980	*A.martynovi* Vishniakova, 1980	4-4-5
			Genus *Asiodiplatys* Vishniakova, 1980	*A.speciosus* Vishniakova, 1980	4-4-5
			Genus *Barbderma*Xing et al., 2016a	*B.oblonguatum* Xing et al., 2016a	?-?-?
			Genus *Longicerciata* Zhang, 1994	*L.mesozoica* Zhang, 1994	5-5-5
				*L.rumpens* Zhang, 1994	5-5-5
			Genus *Microdiplatys* Vishniakova, 1980	*M.campodeiformis* Vishniakova, 1980	4-4-5
				*M.oculatus* Vishniakova, 1980	4-4-5
				*M.perfectus* Vishniakova, 1985	4-4-5
			Genus *Perissoderma*Xing et al., 2016b	*P.triangulum* Xing et al., 2016b	5-5-5
			Genus *Protodiplatys* Martynov, 1925	*P.fortis* Martynov, 1925	4-4-5
				*P.gracilis* Vishniakova, 1980	4-4-5
				*P.mongoliensis* Vishniakova, 1986	4-4-5
			Genus *Sinoprotodiplatys*Nel et al., 2012	*S.ellipsoideuata* Xing et al., 2016a	5-5-5
				*S.zhangi* Nel et al., 2012	5-5-5
		Family Turanoviidae Engel, 2003 (Turanodermaptera Engel, 2019)	Genus *Turanovia* Vishniakova, 1980	*T.incompleta* Vishniakova, 1980	?-?-?

*Note that Whalley (1985) indicated that he could not determine the number of tarsomeres, stating that, “It is not possible to count the exact number of tarsal segments, of which there are certainly four and may well be five.” Thus, we can only state that there are at least four tarsomeres, but it could be fully pentamerous.

Herein we describe a new genus and species of Protodiplatyidae and a new species of Dermapteridae, both preserved in the Middle Jurassic Jiulongshan Formation of Inner Mongolia Province, China. This discovery increases the diversity of Archidermaptera and complements our limited understanding of this suborder.

## Materials and methods

Three specimens were collected from the Middle Jurassic Jiulongshan Formation at Daohugou Village, Ningcheng County, Inner Mongolia, northeastern China. The age of the fossil deposit is approximately 164–165 Ma (Chen et al. 2004; Ren et al. 2010; Gao et al. 2021; Yang et al. 2021), within the Callovian stage of the later Middle Jurassic. The material is housed in the Key Lab of Insect Evolution and Environmental Changes, the College of Life Sciences, Capital Normal University, Beijing (CNU; Dong Ren, Curator). Specimens were examined using a Leica M205C dissecting microscope, with ethanol added to help improve clarity and contrast with the surrounding matrix, thereby aiding the identification of fine details and the preparation of photographs. The detailed and enlarged photos were taken using a Nikon SMZ 25 microscope with a Nikon DS-Ri 2 digital camera. Line drawings were prepared using Adobe Illustrator CC and Adobe Photoshop CS5 graphics software. The higher classification followed herein is that of Engel and Haas (2007), and the morphological terminology employed in this paper is based on that of Giles (1963), Günther and Herter (1974).

## Systematic palaeontology

### Order Dermaptera de Geer, 1773

**Suborder Archidermaptera Bey-Bienko, 1936**

#### Family Protodiplatyidae Martynov, 1925

##### Genus Aneuroderma

Taxon classificationAnimaliaDermapteraProtodiplatyidae

Xiong, Engel & Ren gen. nov.

5F535F40-C707-558F-958C-4FB1C37545B4

http://zoobank.org/1A4122EC-F912-4D76-9D89-A581796CF071

###### Diagnosis.

Moderate-sized earwigs, with numerous setose and distinctively sculptured (densely punctate-granulose throughout, particularly on head and thorax). Head broad, nearly as wide as anterior border of pronotum, posterior margin nearly straight. Antenna with 20 antennomeres; scape robust and slightly broader than remaining antennomeres; pedicel slightly longer than wide; all flagellomeres longer than wide. Compound eyes large and situated at posterior temples; ocelli absent. Dorsal surface without Y-shaped ecdysial cleavage scar. Pronotum approximately oval, anterior and posterior margins subequal in width, lateral margin convex and rounded. Tegmina without longitudinal veins; tegmina and squamata covering abdominal segment II. Legs with abundant short setae; femora carinulate; all tarsi pentamerous (i.e., tarsal formula 5-5-5 rather than the 4-4-5 of some genera); pretarsal claws simple. Female with exposed ovipositor. Pygidium small. Cerci filiform and long, with about 30 cercomeres.

###### Etymology.

The generic name is a combination of the Greek prefix *a*– (*ᾰ*–, alpha privativum designating negation), *neûron* (*νεῦρον*, meaning, “nerve”), and *dérma* (*δέρμᾰ*, genitive *dérmatos*, meaning, “skin” – an allusion to the leathery tegmina and from which the ordinal name is derived, Dermaptera literally meaning, “skin wings”), referencing the absence of tegminal venation, a rare feature among Archidermaptera. The gender of the name is neuter.

##### Aneuroderma oiodes

Taxon classificationAnimaliaDermapteraProtodiplatyidae

Xiong, Engel & Ren, gen. et sp. nov.

E8859788-F6C2-54C8-96AB-1719F8767663

http://zoobank.org/3AE3DBE5-7926-461C-866C-EE41EBDB272A

Figs 1 , 2 , 3

###### Diagnosis.

As for the genus (*vide supra*).

###### Type material.

**Holotype**, a completely preserved female, CNU-DER-NN2021003C/P; **paratype**, CNU-DER-NN2021004C/P. All type material deposited in the College of Life Sciences, Capital Normal University, Beijing, China.

###### Locality and horizon.

Jiulongshan Formation (Middle Jurassic); Daohugou Village, Wuhua Township, Ningcheng County, Inner Mongolia, China.

###### Description.

**Figure 1.**
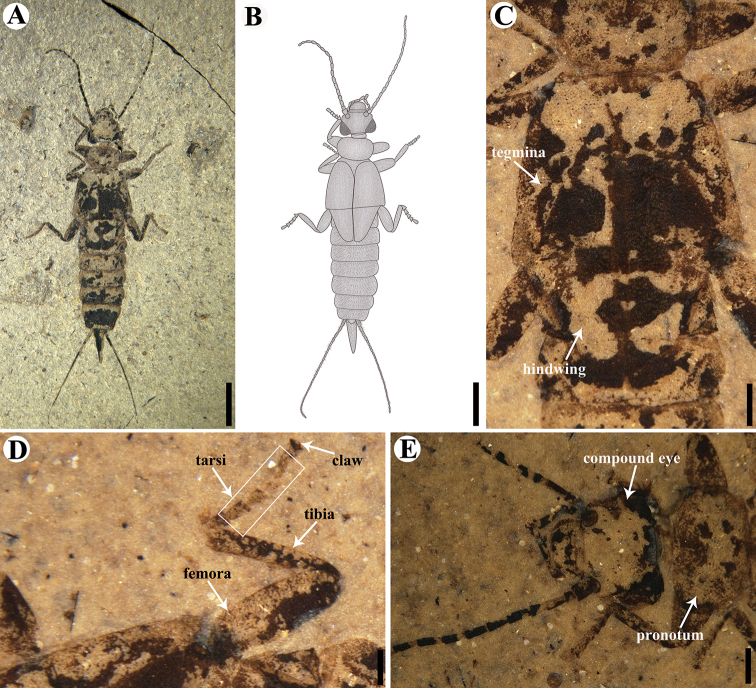
Holotype of *Aneurodermaoiodes* gen. et sp. nov. CNU-DER-NN2021003C. **A** Photograph of dorsal aspect **B** line drawing of dorsal aspect **C** dorsal view of tegmina and squamata of hind wings **D** anterior lateral (prolateral) view of right midleg **E** dorsal aspect of head. Scale bars: 2.0 mm (**A, B**); 0.5 mm (**C, D, E**). The tegmina and squamata of the hind wings in **C**, the right midleg in **D**, and the head in **C** are photographed under ethanol.

**Figure 2.**
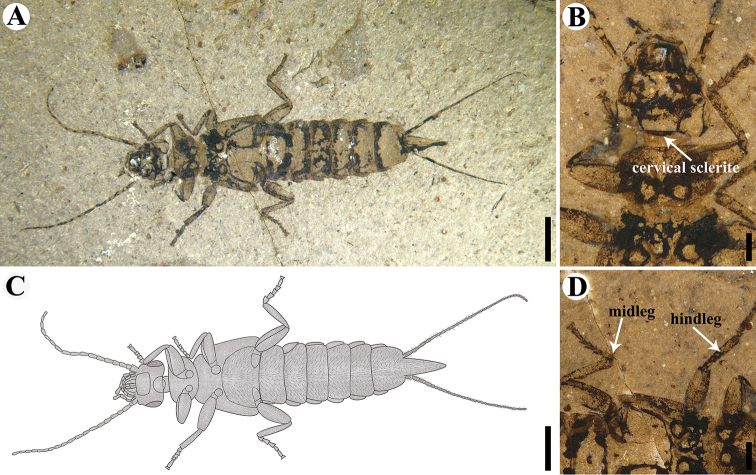
Holotype of *Aneurodermaoiodes* gen. et sp. nov. CNU-DER-NN2021003P. **A** Photograph of ventral aspect **B** ventral view of head **C** line drawing of ventral aspect **D** ventral view of midleg and hindleg. Scale bars: 2.0 mm (**A, C**); 0.5 mm (**B, D**). The head in **C** and the right midleg and hindleg in **D** are photographed under ethanol.

Adult female, preserved in both dorsal and ventral aspects. Body with numerous setae and distinctively sculptured (densely punctate-granulose throughout, particularly on head and thorax). Total length as preserved (excluding antennae, ovipositor, and cerci) about 10.75 mm. Head medial length from clypeal apex to posterior border 1.57 mm, maximum width (across level of compound eyes) 1.56 mm, prognathous; maxillary palpus pentamerous, ca 1.33 mm long (Fig. 1E). Antennal length 5.2 mm, with 20 elongate antennomeres; scape thick, broader than remaining antennomeres, longer than wide, length 0.31 mm, apical width 0.22 mm; pedicel shortest, length 0.17 mm; flagellomeres longer than wide and distally becoming tapered. Compound eyes large and prominent, located near posterior margin of head, compound eye length 0.72 mm; distance between compound eyes distinctly longer than compound eye length (Fig. 1E). Ocelli absent. Pronotum approximately oval and almost as broad as posterior margin of head, medial length 1.12 mm, maximum width 1.71 mm; anterior margin 1.56 mm wide, posterior margin 1.53 mm wide, anterior margin nearly straight, posterior margin slightly convex and lateral margins convexly rounded. Tegmina well developed, without venation, length 2.41 mm, maximum width 1.39 mm, lateral margins arc-shaped, posterior margins truncate, squamata extending well beyond tegminal apex, tegmina and squamata covering abdominal terga I and II (Fig. 1C). Femora compressed and ventrally carinulate; tibiae elongate, slender, almost as long as femora; all tarsi pentamerous (Fig. 2D). Pretarsal claws present and simple; arolium absent (Fig. 1D). Abdomen cylindrical, with dense, soft, short setae, lateral margins relatively convex, almost all segments wider than long with apical margins straight, abdominal length as preserved (excluding cerci) 5.38 mm, maximum width 2.41 mm. Abdomen distally with external ovipositor, length 1.64 mm. Pygidium small. Cerci 5.33 mm long, longer than one-half abdominal length, with ca 30 elongate cercomeres, margins with abundant short setae.

###### Etymology.

The specific epithet is the Greek neuter adjective *ōiôdes* (*ᾠῶδες*, meaning, “oval” or “egg-like”), as a reference to the ovoid pronotum.

###### Remarks.

The new genus is placed within Protodiplatyidae on the basis of the characteristic filiform antenna with 17–23 antennomeres; pedicel and flagellomere I subequal in size; pentamerous metatarsus; cerci elongate, slender, and multimerous; and externalized ovipositor in females.

Key diagnostic characters of *Aneuroderma* gen. nov. are summarized in Table 2 and compared with those of nine genera of Protodiplatyidae. *Aneuroderma* gen. nov. can be distinguished from *Archidermapteron* Vishniakova, 1980 by the posterior margin of head as wide as anterior border of pronotum, 20 antennomeres, tegmina without longitudinal veins, and hind wings extending to apex of abdominal segment II, pygidium small, 30 cercomeres, and a cercus/body ratio of 0.5. By contrast, *Archidermapteron* has the head narrower than the pronotum, the tegmina with longitudinal veins, hind wings extending beyond abdominal segment IV, pygidium transverse and trapeziform, and 40 cercomeres that together are slightly shorter than the body. *Aneuroderma* gen. nov. differs from *Longicerciata* Zhang, 1994 by the latter with the head broader than the pronotum, 26 antennomeres, at least 36 cercomeres, and a cercus/body ratio of 1. *Aneuroderma* gen. nov. differs from *Barbderma*Xing et al., 2016a in the number of antennomeres (20 instead of 19), and the hind wings extending to the apex of abdominal segment II, instead of segment I. *Aneuroderma* gen. nov. is similar to *Asiodiplatys* Vishniakova, 1980 in that the tegmina lack longitudinal veins and the hind wings extend to the apex of abdominal segment II, but the former differs from the latter in only 20 antennomeres (vs. 22 antennomeres), the posterior margin of the pronotum straight (vs. pronotum with shallow, broad notch anteriorly), and 30 cercomeres (vs. 40 cercomeres). *Aneuroderma* gen. nov. can be separated from *Abrderma*Xing et al., 2016b, *Microdiplatys* Vishniakova, 1980, *Perissoderma*Xing et al., 2016b, and *Protodiplatys* Martynov, 1925, by the following traits: (1) absence of longitudinal veins in tegmina (present in the aforementioned genera), and (2) 20 antennomeres (vs. 17 to 19 in the other genera). The new genus is readily differentiated from *Sinoprotodiplatys*Nel et al., 2012 by the posterior margin of the head and anterior border of the pronotum equal in width (head narrower than pronotum in the latter), 20 antennomeres, and 30 cercomeres (18 antennomeres and 20 cercomeres in the latter). Lastly, the distinctive punctate sculpture of the new genus is quite distinctive among several Archidermaptera.

**Table 2.** Summary and comparisons of key characters for several described genera of Protodiplatyidae. Interrogative marks (?) indicate data unavailable.

Genus	Head	Number of antennomeres	Pronotum	Tegmina	Pygidium	Cerci (ratio lengths cercus/body, cercomeres)
*Abrderma*	Broader than pronotum	17–19	Elliptical, anterior and posterior margins subequal in width	With venation, reaching second abdominal segment	Small	0.2, ?
*Archidermapteron*	Narrower than pronotum	17–19	Reniform, broad notch anteriorly	With venation, reaching fourth abdominal segment	Transverse, trapeziform	1.0, at least 40
*Asiodiplatys*	Narrower than pronotum	22	With shallow, broad notch anteriorly	Without venation, not reaching second abdominal segment	?	0.5, 40
*Barbderma*	?	19	Oblong or trapezoidal, anterior and posterior margins subequal in width	With venation, reaching first abdominal segment	Small	0.42, ?
*Longicerciata*	Broader than pronotum	26	Transverse, anterior margin wider than posterior margin	Without venation, reaching fourth abdominal segment	?	1.0, at least 36
*Microdiplatys*	Broader than pronotum	19	Transverse	With venation, reaching fourth abdominal segment	Not protruding	1.0, at least 36
*Perissoderma*	Narrower than pronotum	17	Elliptical, anterior margin wider than posterior margin	With venation, reaching third abdominal segment	Small	0.5, ?
*Protodiplatys*	Narrower than pronotum	17–18	Transverse, notch in front, broadly rounded posteriorly	With venation, reaching fourth abdominal segment	Transverse, trapeziform	0.5, no more than 40
*Sinoprotodiplatys*	Narrower than pronotum	18	Anterior and posterior margins subequal in width	Without venation, reaching fourth abdominal segment	?	0.8, 20
*Aneuroderma* gen. nov.	As wide as pronotum	20	Oval, anterior and posterior margins subequal in width	Without venation, reaching second abdominal segment	Small	0.5, 30

**Figure 3.**
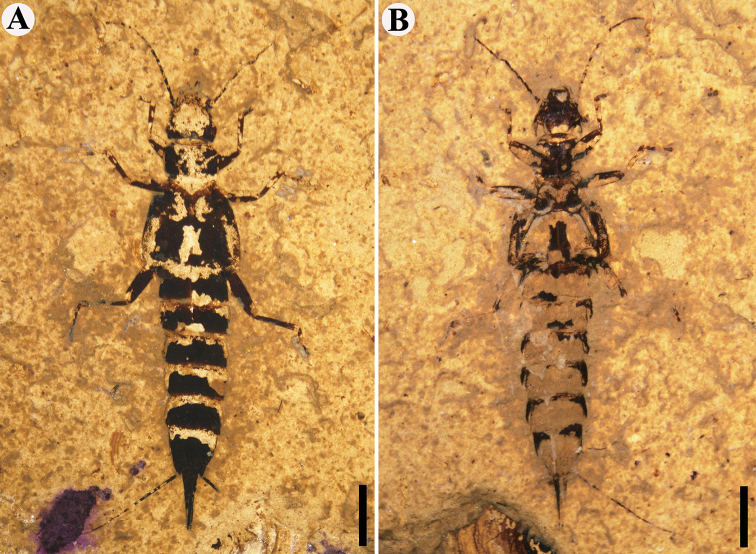
Paratype of *Aneurodermaoiodes* gen. et sp. nov. CNU-DER-NN2021004C/P. **A** Photograph of dorsal aspect **B** photograph of ventral aspect. Scale bars, 2.0 mm (**A, B**). The body in **A** and **B** are photographed under ethanol.

#### Family Dermapteridae Vishniakova, 1980

##### Genus Sinopalaeodermata

Taxon classificationAnimaliaDermapteraDermapteridae

Zhang, 2002

0A3A9F5C-4ECF-5E24-A136-01941B7EA34E

Sinopalaeodermata Zhang, 2002: 351. Type species: Sinopalaeodermataneimonggolensis Zhang, 2002, *nomen imperfectum* [recte neimonggolense], by original designation.

###### Emended diagnosis

[modified from Zhang (2002)]. Moderate-sized earwigs, with short and fine pubescence; head triangular, length subequal to width, mandibles denticulate; antenna filiform, with at least 19 antennomeres (as noted by Zhang (2002)), scape stout and enlarged, pedicel rather small, clearly shorter than scape and flagellomere I; ocelli present; neck prominent, divided into anterior and posterior cervical sclerites (‘blattoid’ neck arrangement). Pronotum transverse, broader than long and about as broad as head. Femora compressed and ventrally carinulate; all tarsi pentamerous, shorter than tibiae; pretarsal claws well developed and bearing broom-shaped arolia. Procoxal cavities positioned close to each other; mesocoxal cavities remote, metacoxal cavities elongate transversely and moderately separated. Tegmina with longitudinal veins strongly developed, main veins of Sc, R, M, and Cu present. Female with a pair of elongate valvulae entirely exposed. Cerci flexible, long, filiform, and multimerous.

**Included species.** Aside from the type species, the genus currently includes *S.concavum* Xiong, Engel & Ren, sp. nov. (*infra*).

##### Sinopalaeodermata concavum

Taxon classificationAnimaliaDermapteraDermapteridae

Xiong, Engel & Ren sp. nov.

B6FC5D80-7AA5-5973-97B2-7288C7E4849D

http://zoobank.org/9759E27B-8954-48A1-B220-57514C01F07F

Figures 4 , 5

###### Diagnosis.

The new species can be distinguished from the type species, *Sinopalaeodermataneimonggolense* (note that the name *Sinopalaeodermata* is neuter, not feminine, as *dérmata* is the neuter nominative plural of *dérma*; and given that the specific epithet is adjectival it must still agree in gender with the generic name) by the relatively straight apical margin of the penultimate sternum (in *S.neimonggolense* the penultimate sternum has a concave margin); the roughly reniform pronotum, with the anterior margin concave medially the posterior margin weakly convex, and lateral margins rounded (in *S.neimonggolense* the pronotum is approximately rectangular, with the anterior margin almost as wide as the posterior margin, and the lateral margins relatively straight and parallel to each other); the tegmina with a more pronounced concave arc marginally at the apex of Rs (in *S.neimonggolense* the margin is more sloping rather than deeply concave); and M does not extend to near the apex of CuA , with CuP terminating more proximal to CuA (even before the tangent with M) (in *S.neimonggolense* M terminates more proximally and CuP extends to the apex of CuA).

**Holotype.** A completely preserved female, CNU-DER-NN2021005C/P, deposited in the College of Life Sciences, Capital Normal University, Beijing, China.

###### Locality and horizon.

Jiulongshan Formation (Middle Jurassic); Daohugou Village, Wuhua Township, Ningcheng County, Inner Mongolia, China.

###### Description.

**Figure 4.**
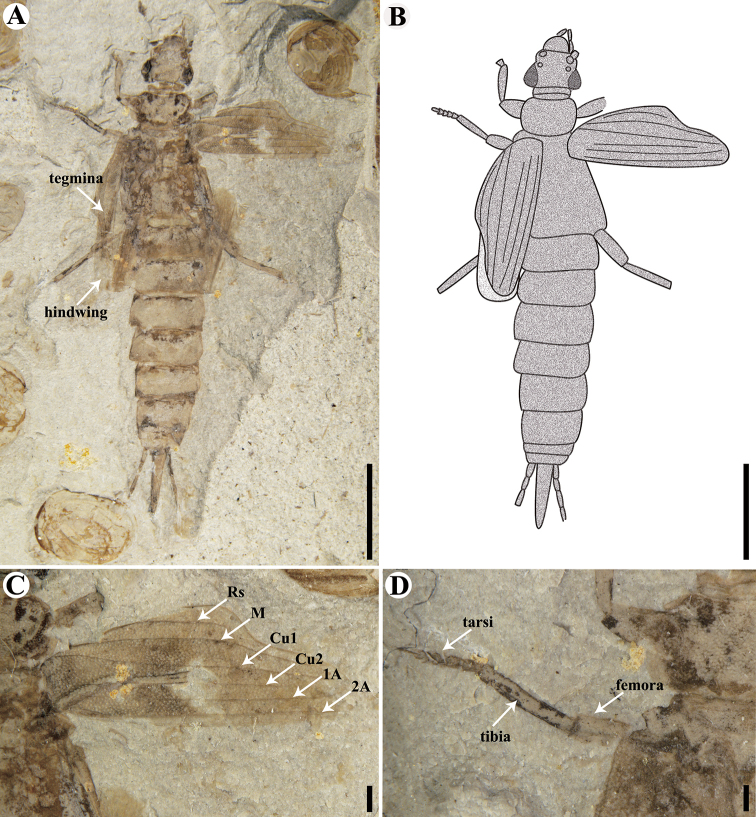
Holotype of *Sinopalaeodermataconcavum* sp. nov. CNU-DER-NN2021005C. **A** Photograph of dorsal aspect **B** line drawing of dorsal aspect **C** dorsal view of right tegmen **D** anterior lateral (prolateral) view of left midleg. Scale bars: 4.0 mm (**A, B**); 0.5 mm (**C, D**).

**Figure 5.**
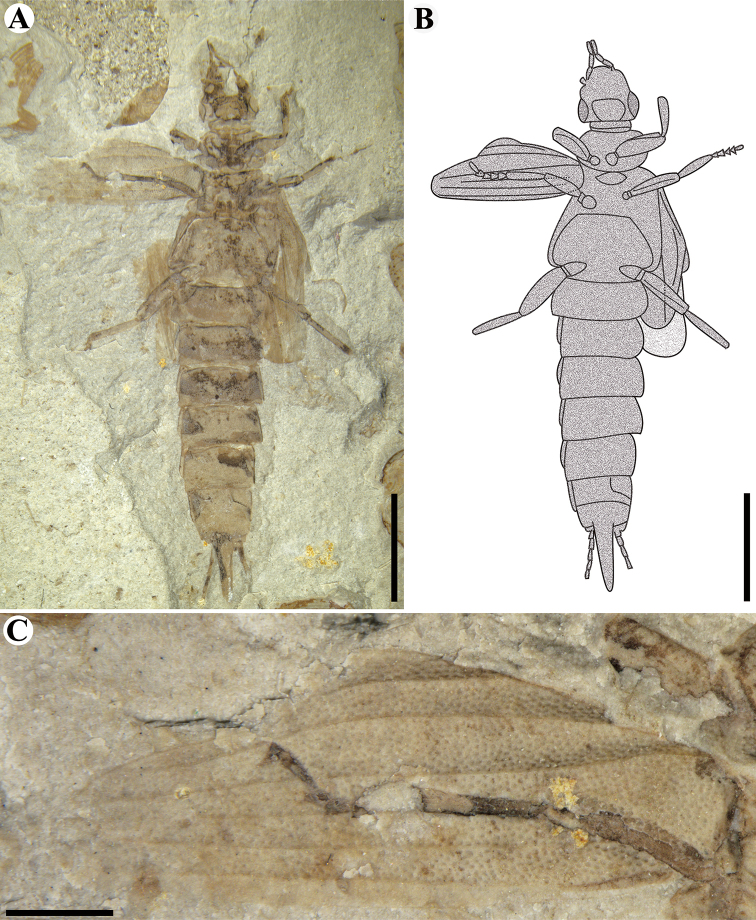
Holotype of *Sinopalaeodermataconcavum* sp. nov. CNU-DER-NN2021005P. **A** Photograph of ventral aspect **B** line drawing of ventral aspect **C** ventral view of right midleg and tegmen. Scale bars: 4.0 mm (**A, B**); 1 mm (**C**).

Adult female, preserved in both dorsal and ventral aspects. Total length as preserved (excluding antennae, cerci, and valvulae) about 18.02 mm. Body with sparse pubescence and punctate. Head medial length from clypeal apex to posterior border 2.08 mm, maximum width (across level of compound eyes) 2.27 mm, triangular. Compound eye large, ovate, located near posterior margin of head; compound eye length 0.91 mm; width between compound eyes 2.58 mm. Ocelli comparatively small. Cervix with large anterior and posterior cervical sclerites, anterior sclerite slightly larger than posterior sclerite. Pronotum approximately reniform, medial length 1.46 mm, maximum width 2.58 mm, anterior width 1.64 mm, posterior width 2.07 mm, anterior margin concave and posterior margin arched, lateral margins convexly rounded. Mesoscutellum large, elliptical, entirely exposed. Tegmina present, not truncated, length 6.73 mm, maximum width 2.55 mm, with medially sinuate anterior (lateral) margin and straight posterior (mesal) margin. Veins simple, Rs curved anterior margin, fading out just before margin; M simple, basally and apically straight, gently curved medially; Cu with two branches (CuA and CuP), CuP terminates proximal to CuA; A1 and A2 simple and straight, running parallel to each other and posterior margin, terminating apically (Fig. 4C). Femora compressed and ventrally carinulate (Fig. 4D); tibiae elongate, slender, and almost as long as femora; tarsi pentamerous, tarsomere IV slightly extending under base of tarsomere V (Fig. 5C). Pretarsal claws present but not well preserved. Abdominal length as preserved (excluding cerci) 9.75 mm, maximum width 3.52 mm; all segments distinctly wider than long, lateral abdominal margins gently convex. Pygidium not evident. Ovipositor exposed, 2.84 mm long. Cerci as preserved only 2.9 mm long, with segments but not clearly preserved.

###### Etymology.

The specific epithet is taken from the Latin adjective *concavus* (meaning, “concave”), in reference to the more pronounced concave margin to the tegmina relative to the type species.

## Discussion

Both of the new species described herein are easily recognized as archidermapterans owing to the pentamerous metatarsi (and more than three pro- and mesotarsomeres); elongate, flexible, and multimerous cerci; and the externalized ovipositor. Jurassic earwigs are known mostly from four assemblages: the Jiulongshan flora of the Middle Jurassic, the England flora of the Early and Late Jurassic, and the Karatau flora of the Late Jurassic (Vishniakova 1980; Zhang 1994, 2002; Zhao et al. 2011; Kelly et al. 2018; Tihelka 2019). Archidermaptera were seemingly the most abundant form of earwigs during these epochs, with 28 species in 21 genera (Table 1), compared to only seven species in five genera of Eodermaptera. Numerous fossil insects and plants have been described from the Jiulongshan Formation (e.g., Ren et al. 2010; Gao et al. 2021), yet earwigs are comparatively rare, with only eight species reported from the locality.

Tarsal formulae have been used to distinguished significant groups of fossil Dermaptera. All Neodmerpatera and Eodermaptera have three tarsomeres (3-3-3), while the number of tarsomeres is greater, where known, among Archidermaptera (Table 1) (Engel 2003). Unfortunately, tarsi are unknown for many fossil genera, particularly among the Dermapteridae, and so general patterns are difficult to determine. Nonetheless, historically the Dermapteridae have been considered to have a 5-5-5 forumula, while Protodiplatyidae include genera with 5-5-5 and 4-4-5 formulae (Engel 2003; Zhao et al. 2011). What remains to be ascertained from a detailed phylogenetic study is whether the variable tarsal formulae among Protodiplatyidae is reflective of paraphyly on the part of this family, or whether it renders Dermapteridae paraphyletic. Unfortunately, currently available specimens and data do not allow for a robust resolution of this difficulty, thus emphasizing the need for further exploration of Jurassic and Late Triassic deposits throughout the world in the hope of recovering more completely preserved material of these lineages and allowing a comprehensive comparison among living and fossil Dermaptera.

## Conclusion

Based on three well-preserved fossil specimens from the Middle Jurassic, we describe a new genus and two new species, *Aneurodermaoiodes* gen. et sp. nov. (Protodiplatyidae) and *Sinopalaeodermataconcavum* sp. nov. (Dermapteridae). We make extensive comparisons with other Archidermaptera and discuss challenges in understanding relationships among these Triassic and Jurassic lineages.

## Supplementary Material

XML Treatment for Aneuroderma

XML Treatment for Aneuroderma oiodes

XML Treatment for Sinopalaeodermata

XML Treatment for Sinopalaeodermata concavum
